# Thoracic Ultrasound for Immediate Exclusion of Pneumothorax after Interventional Bronchoscopy

**DOI:** 10.3390/jcm9051486

**Published:** 2020-05-15

**Authors:** Stephan Eisenmann, Jane Winantea, Rüdiger Karpf-Wissel, Faustina Funke, Elena Stenzel, Christian Taube, Kaid Darwiche

**Affiliations:** 1 Department of Pulmonary Medicine, University Hospital of Halle-Wittenberg, 06120 Halle, Germany; 2 University Hospital of Essen, West German Lung Center, Ruhrlandklinik, 45239 Essen, Germany; jane.winantea@rlk.uk-essen.de (J.W.); ruediger.karpf-wissel@rlk.uk-essen.de (R.K.-W.); faustina.funke@rlk.uk-essen.de (F.F.); christian.taube@rlk.uk-essen.de (C.T.); kaid.darwiche@rlk.uk-essen.de (K.D.); 3 Department of Diagnostic and Interventional Radiology, University Hospital of Essen, 45147 Essen, Germany; elena.stenzel@uk-essen.de

**Keywords:** pneumothorax, lung ultrasound, interventional bronchoscopy

## Abstract

Background. Pneumothorax is a common side effect in interventional pulmonology. The ideal moment for detection with chest X-ray or ultrasound has not yet been defined. Earlier studies demonstrated the utility of performing these tests with a certain delay, which always results in a potentially dangerous gap. Methods. We prospectively enrolled patients with pulmonary interventions at increased risk of pneumothorax. Thoracic ultrasound was performed immediately after the intervention and at the moment of chest X-ray with a delay up to two hours. Results: Overall, we detected four pneumothoraxes in 115 procedures. Sensitivity, specificity, positive predictive value, negative predictive value and accuracy were 75%, 100%, 100%, 99%, 99% for ultrasound and 75%, 90%, 21%, 99% und 89% for chest X-ray respectively. All pneumothoraces requiring chest tube were sufficiently detected by both methods. Conclusion. Thoracic ultrasound when performed immediately can more accurately exclude pneumothorax after interventional bronchoscopy when compared to chest X-ray. Further ultrasound examinations are unnecessary.

## 1. Background

Interventional bronchoscopy procedures are linked to an increased incidence of pleural injury leading to a pneumothorax. Early and sufficient management is crucial to avoid cardiopulmonary deterioration. 

Chest x-ray is still widely established to document pulmonary integrity. However, sensitivity, specificity and diagnostic yield of a chest X-ray are low [1,2] and additional tools are needed to support or replace post-interventional chest x-rays [3]. 

Thoracic ultrasound (TUS) is an appropriate tool for the exclusion of a pneumothorax and has been widely researched in critical care and emergency settings [4,5]. It is, therefore, mentioned as a comparable tool when compared to X-ray for the exclusion of pneumothorax and, e.g., is mentioned in the latest German guideline [6]. Exclusion of a pneumothorax in the supine position with overall detection of either lung sliding or lung pulse, while the visualization of a lung pulse confirms a pneumothorax [7]. Usually radiological or sonographic check is planned after an indefinite delay, which is in real life often difficult to organize. We now wanted to evaluate TUS as an immediate examination tool to diagnose or exclude a post-interventional pneumothorax and also compare the use of a linear and convex ultrasound transducer in the same patient.

## 2. Methods

This is a prospective and single center feasibility study of enrolled patients scheduled for a procedure with an assumed risk of post-bronchoscopic pneumothorax: transbronchial lung biopsy with forceps (TBB), transbronchial needle aspiration (TBNA) or transbronchial cryobiopsy (cTBB), and bronchoscopic lung volume reduction (BLVR) procedures with endobronchial valves (EBV, Pulmonx^®^, Redwood City, CA, USA and IBV, Olympus^®^, Tokyo, Japan) or Lung Volume Reduction Coil (PneumRx, Inc., Mountain View, CA, USA). All prospective patients with the mentioned procedures at age > 18 years were enrolled after signing the informed consent. Exclusion from the study occurred in the absence of informed consent, and pediatric patients were also not included.

Bronchoscopies were carried out at the Ruhrlandklinik Essen, a German tertiary university center. The interventions were executed using total intravenous anesthesia (TIVA) and with a combination of rigid bronchoscopy (Storz^®^, Tuttlingen, Germany) under jet ventilation and flexible bronchoscopy (Olympus®, different flexible bronchoscopes). After the intervention and extubation, all patients were transferred to the intermediate care unit and after 2–4 h, to the general ward.

Chest X-ray was performed in inspiration and supine position by every patient two hours after BLVR, and, in the case of a pneumothorax or uncertain diagnosis and clinical stability, 24 h later in an upright position. Radiological examinations were analyzed by a radiologist who was blinded to the result of the ultrasound examination. Radiologic categories were set as definitive pneumothorax, possible pneumothorax (or uncertainty), and exclusion of pneumothorax.

TUS was performed bilaterally either with a 20 MHz linear probe or a 7.5 MHz convex probe (Philips CX 50^®^, Best, The Netherlands) in the supine position at three consecutive points:

- Postprocedural under jet ventilation.
- Under spontaneous breathing but still in the bronchoscopy room.
- Two hours after the intervention, together with X-ray.

**Figure 1:**
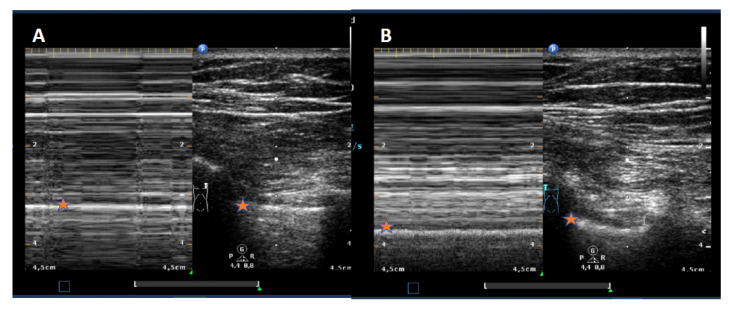
TUS with M-Modus showing the absence of lung sliding (**A**, “barcode sign” with pneumothorax) and presence of lung sliding (**B**, “seashore-sign”, no pneumothorax). The difference could be noted in the M-Modus-parts below the star-marked white line, that reflects a regular (**B**) or missing sliding pleural line, leading to repetitive horizontal lines in the case of a pneumothorax in part (**A**).

No specific thorax preset was used. The TUS examination was done by one experienced ultrasound examiner as described earlier [7]: it started parasternal at the adjacent intercostal spaces (ICR) at the medio clavicular line (MCL). Pleural integrity was assumed if all three following features were detectable: pleural sliding, B-Lines, and lung pulse (M-Modus). In the absence of one out of these items, the lung point, as the proof of pneumothorax, was searched for following the ICR laterally and marked with a strip placed on the skin. If a unilateral lung point was not detectable, a total pneumothorax was assumed. Absence of lung sliding was documented by addition of M-mode with the typical barcode-sign [7], as shown in Figure 1.

Definitive pneumothorax was defined as detectable with at least one of the modalities and clinical signs. A definitive exclusion of a pneumothorax was assumed with a secure exclusion of all modalities and lacking clinical evidence of a pneumothorax.

In the case of pneumothorax detection by any of the methods and clinical stability, conservative management and radiologic and ultrasound control were scheduled. In the case of uncertainty in TUS and X-ray examination, and clinical necessity, a computed tomography (CT) was scheduled. If a pneumothorax was proven by any of the methods, and if clinically necessary, a chest tube was placed.

Data were collected by dedicated case report form (CRF) and statistical analysis was performed with SPSS (IBM®, version 26, Armonk, NY, United States). The study was approved as a prospective non-interventional study by the institutional review board (IRB) of the University of Essen, Germany (No. BO 15-6572) and at the German Registry of Clinical Studies (Universal Trial No. U1111-1176-9066, www.drks.de). As an investigator-initiated trial, there was no external funding.

## 3. Results

We prospectively enrolled 115 patients (48 with BLVR procedures, 67 with TBB procedures; patients’ details are reflected in Table 1) into the study between December 2015 and August 2016.

The BLVR cohort consisted of 39 patients with valves (only EBV, no IBV) and 9 patients with coils. In the valve subgroup, 33 patients (84.6%) developed an atelectasis within 72 h.

The TBB cohort consisted of 36 patients with conventional TBB and 31 patients with cTBB. 

Overall four pneumothoraxes occurred in the early postinterventional period, thereof two after TBB (middle lobe and right lower lobe), one after cTBB (left upper lobe) and one pneumothorax ex vacuo 24 h after valve placement in the right upper lobe. Two patients (one TBB, one cTBB) required a chest tube for less than seven days, while the other two patients could be managed conservatively.

One additional patient developed a delayed and symptomatic pneumothorax after valve therapy, which was diagnosed by TUS and X-ray, but outside the study period. This patient was initially analyzed as normal since a pneumothorax did not happen within the first 24 h. Four more EBV patients developed a delayed pneumothorax but were not investigated by TUS at this moment.

Initial ultrasound under controlled jet ventilation showed two true positives, but no false positive pneumothoraxes. This led to a sensitivity, specificity, positive predictive value (PPV), negative predictive value (NPV) and accuracy of 50%, 100%, 100%, 98% and 98% respectively. 

Ultrasound under spontaneous breathing in the operating room (OR) showed three positive and no false negative results. The lung point was visible in these three patients. The detection rate did not rise with a further examination after two hours. This results in a sensitivity, specificity, PPV, NPV and accuracy of 75%, 100%, 100%, 99% and 99% respectively for the early TUS examination (Table 2). In the two patients requiring chest tube treatment, the initial lung point position changed laterally, assuming an increasing pneumothorax volume. This corresponded to clinical impairment with dyspnea and chest pain. The initially missed pneumothorax was additionally detected 24 h after EBV placement by X-ray, showed no clinical impairment and was defined as pneumothorax ex vacuo. This pneumothorax was neither described in the initial X-ray nor in the ultrasound examinations. With formation of the ex vacuo pneumothorax, the lung point was also visible by TUS after 24 h. There was no difference between the two ultrasound transducers (*p* > 0,05, data not shown).

Chest X-ray in the supine position after two hours showed 14 suspicious results and included three true and 11 uncertain cases. Nine of the 11 uncertainties resulted from inadequate X-ray performance, usually missing details of the thorax anatomy that resulted in uncertain diagnosis. The one patient with pneumothorax after EBV placement was initially categorized as inconspicuous with a developing atelectasis in the left upper lobe, but the pneumothorax was proven in the upright control after 24 h. The lung point was not visible in the post-interventional study ultrasound, but was detectable after 24 h, showing that there was no relevant atelectasis formation at this early moment. All further uncertain results showed no pathological finding in upright X-ray. CT was not performed. This resulted in a sensitivity, specificity, PPV, NPV and accuracy of 75%, 90%, 21%, 99% and 89% respectively (Table 3).

## 4. Discussion

Thoracic ultrasound when performed under spontaneous breathing can accurately diagnose and exclude a postinterventional pneumothorax. TUS can be done instantly after the termination of the endoscopy in spontaneous breathing. The results are comparable to previous reports with immediate and delayed control after another two hours, where TUS was already reported as a feasible method in TBB and cTBB in different patient cohorts [8,9,10]. However, no study has investigated comparability of TUS in either an immediate or delayed situation. When compared to initial chest X-ray, the difference in accuracy shows TUS again to be the superior method. 

All initial pneumothoraxes were connected with TBB procedures and instantly detectable under spontaneous breathing. When performed under jet ventilation, TUS would miss one out of three pneumothoraxes. Jet ventilation is known to reduce visibility of pleura sliding and therefore potentially leading to misinterpretation [11]. However, in our study, under jet ventilation there was no false positive finding but a decreased sensitivity. This might reflect the learning curve of the examiner who had not been routinely familiar with TUS under jet ventilation previously. This should be considered when planning an optimal point in time for the ultrasound examination.

If EBV treatment was not part of the study, the sensitivity of post-interventional TUS could have been as high as 100%. However, it is debatable if this single ex vacuo pneumothorax could have been detected early after intervention. Later on, the pneumothorax was diagnosed with X-ray and TUS, and one additional patient with a delayed pneumothorax was accurately diagnosed by both modalities outside of the study.

This leads to the assumption that a qualified ultrasound examination immediately after endoscopic procedures can securely replace delayed chest x-ray or ultrasound examination to diagnose and exclude a pneumothorax. No additional ultrasound is necessary in the case of clinical stability and initial exclusion of a pneumothorax.

After TBB, a pneumothorax occurs in 3–5% of the patients. The incidence was higher in our cohort, probably due to the integration of patients with cTBB. The incidence of a pneumothorax after valve therapy and atelectasis is reported as 25% [12], but was much lower in our cohort even though we had a high rate of atelectasis development. However, we just focused on early pneumothorax detection. Four additional patients after ELVR suffered pneumothorax later on that was not observed by ultrasound due to missing TUS experience at that moment. If there is a clinical possibility for a delayed pneumothorax occurrence, as in after atelectasis development due to EVLR with valves, ultrasound might be as sensitive as X-ray for its detection at any later moment.

After ELVR with valves, pneumothorax often occurs in a vulnerable period within 72 h after valve placement [12]. This is in contrast to a pneumothorax that occurs after TBB, where the moment of injury is clearly defined. The visibility of a lung point after ELVR, as seen in one of our patients, and in the absence of clinical worsening could be a sign of a pneumothorax ex vacuo and connected with the development of atelectasis. Without clinical symptoms there is no need for a chest tube. In combination with clinical symptoms of pneumothorax, X-ray might be dispensable for the acute diagnosis of pneumothorax, even after BLVR, regardless of the period to the intervention.

Pneumothorax detection and exclusion was accurately feasible with different transducers. Recently it had been proven that pneumothorax detection does not require a specific transducer in surgically induced pneumothorax [13]. Here we demonstrate the equal suitability of either a linear or a convex transducer in the exclusion of pneumothorax, which is a routinely addressed issue in daily practice.

Chest X-ray in the supine position led to a high number of uncertain diagnoses, especially due to the generation of a high number of incorrect pictures. This is important because in typical clinical situations chest X-ray is often performed in the intensive care unit (ICU) or radiology suite, with a delay after the intervention, in the supine position and with a reduced picture quality. X-ray analyzation will then be accomplished after a further delay by a radiologist, which leads to repetitive examinations and a potentially dangerous gap for the patients. The one pneumothorax in our study that was missed by the radiologist but detected by ultrasound under spontaneous breathing led to a tension pneumothorax. This shows that ultrasound in the case of pneumothorax cannot predict a fatal course or replace clinical monitoring. But in comparison to a delayed X-ray, TUS can provide immediate information on whether potential complications may occur and therefore influences the initial post-interventional monitoring with a focus on increasing patient safety.

Asymptomatic pneumothorax was reported to be small and even invisible in routine chest X-ray, assuming that chest X-ray might be dispensable in asymptomatic patients, but helpful in symptomatic patients [1,2]. Due to increasing workload in daily practice, immediate information after bronchoscopy is useful for cost effective management without repetitive follow up visits to explore potential symptoms.

This study has some limitations. The ultrasound examination was performed by an experienced physician, so it might be questionable as to whether the results would be the same by less experienced physicians. Due to the considerable learning curve of thoracic ultrasound, possible misinterpretation should be kept in mind when starting with the method. The number of patients with pneumothorax in this study is too low to define TUS as the gold standard for the detection of pneumothorax at immediate inspection. This might be addressed in a cohort with a higher relative risk. But as pneumothorax is a known complication in 3–5% of cases after TBB [2], TUS is reliably useful to distinguish patients without a risk of post-procedural complications, helps simplifying the postprocedural monitoring and avoids unnecessary X-ray-exposition. This is clinically important as pneumothorax exclusion as a main indication is still not widespread in Germany [14].

## 5. Conclusions

In conclusion, TUS can accurately exclude a pneumothorax immediately after bronchoscopy independently from the ultrasound transducer available. TUS should therefore become an essential part of the interventional pulmonary instrumentation. A delay of two hours between intervention and TUS, as published earlier, is neglectable.

## Figures and Tables

**Table 1:** Patients demographic and procedure details. Data are *n* or mean (standard deviation).

Patients Demographic and Procedure Details	Data
Age	62.7 (9.9)
Height in cm	170.1 (9.8)
Weight in kg	72.2 (17)
Women/Men	61/54
Endobronchial valve therapy (EBV)	39
Lunge Volume Reduction Coils	9
Transbronchial forceps biopsy (TBB) for ILD	11
Transbronchial cryoprobe biopsy (cTBB) for ILD	27
Transbronchial biopsy for tumor diagnosis	29

**Table 2:** Thoracic ultrasound (TUS) to detect pneumothorax immediately and after two hours, there was no difference for immediate TUS and after two hours.

Final Diagnosis	Thoracic Ultrasound		Total
	Positive	Negative	
Pneumothorax	3	1	4
No pneumothorax	0	111	111
Total	3	112	115

**Table 3:** Chest X-ray after two hours.

Final Diagnosis	X-ray Analyzation		Total
	Positive	Negative	
Pneumothorax	3	1	4
No pneumothorax	11	100	111
Total	14	101	115

## References

[1] Frazier W.D., Pope T.L., Findley L.J. (1990). Pneumothorax following transbronchial biopsy. Low diagnostic yield with routine chest roentgenograms. Chest.

[2] Izbicki G., Romem A., Arish N., Cahan C., Azulai H., Chen-Shuali C., Tennenhaus E., Bar-Yosef Z., Zlotkevich E., Rokach A. (2016). Avoiding Routine Chest Radiography after Transbronchial Biopsy is Safe. Respiration.

[3] Alrajab S., Youssef A.M., Akkus N.I., Caldito G. (2013). Pleural ultrasonography versus chest radiography for the diagnosis of pneumothorax: Review of the literature and meta-analysis. Crit. Care.

[4] Alrajhi K., Woo M.Y., Vaillancourt C. (2012). Test Characteristics of Ultrasonography for the Detection of Pneumothorax: A Systematic Review and Meta-analysis. Chest.

[5] Mayo P.H., Copetti R., Feller-Kopman D., Mathis G., Maury E., Mongodi S., Mojoli F., Volpicelli G., Zanobetti M. (2019). Thoracic ultrasonography: A narrative review. Intensive Care Med..

[6] Schnell J., Beer M., Eggeling S., Gesierich W., Gottlieb J., Herth F.J.F., Hofmann H.S., Jany B., Kreuter M., Ley-Zaporozhan J. (2019). Management of Spontaneous Pneumothorax and Post-Interventional Pneumothorax: German S3 Guideline. Respiration.

[7] Volpicelli G. (2011). Sonographic diagnosis of pneumothorax. Intensive Care Med..

[8] Kumar S., Agarwal R., Aggarwal A.N., Gupta D., Jindal S.K. (2015). Role of Ultrasonography in the diagnosis and Management of Pneumothorax Following Transbronchial Lung Biopsy. J. Bronchol. Interv. Pulmonol..

[9] Bensted K., McKenzie J., Havryk A., Plit M., Ben-Menachem E. (2018). Lung Ultrasound after transbronchial biopsy for pneumothorax screening in post-lung transplant patient. J. Bronchol. Interv. Pulmonol..

[10] Matus I., Raja H. (2019). Protocolized thoracic ultrasonography in transbronchial lung cryobiopsies. J. Bronchol. Interv. Pulmonol..

[11] Lichtenstein D.A. (2005). General Ultrasound in the Critically Ill.

[12] Slebos D.J., Shah P.L., Herth F.J., Valipour A. (2017). Endobronchial Valves for endoscopic lung volume Reduction: Best Practice Recommendations from Expert Panel on Endoscopic Lung Volume Reduction. Respiration.

[13] Ketelaars R., Gülpinar E., Roes T., Kuut M., van Geffen G.J. (2018). Which ultrasound transducer type is best for diagnosing pneumothorax?. Crit. Ultrasound J..

[14] Berlet T., Fehr T., Merz T.M. (2014). Current practice of lung ultrasonography (LUS) in the diagnosis of pneumothorax: A survey of physician sonographers in Germany. Crit. Ultrasound J..

